# Mendelian randomization study showed no causality between metformin treatment and polycystic ovary syndrome

**DOI:** 10.1371/journal.pone.0321380

**Published:** 2025-04-03

**Authors:** Liting Lin, Huan Shen, Yanbin Wang

**Affiliations:** Reproductive Medical Center, Peking University People’s Hospital, Peking University, Beijing, China; Universitas 17 Agustus 1945 - Jakarta, INDONESIA

## Abstract

**Background:**

Despite previous clinical studies providing some evidence of an association between metformin treatment and polycystic ovary syndrome(PCOS), these findings remain controversial. To investigate whether the association reflect causality, a two-sample Mendelian randomization (MR) method was conducted.

**Methods:**

Data from genome-wide association studies were analyzed, with the exposure factor being metformin and the outcome variable being PCOS. The inverse variance weighted(IVW) was used as the primary method for MR analysis. In addition, MR–Egger, weighted median, heterogeneity tests, and sensitivity analyses were performed.

**Results:**

The initial and validation MR analyses indicated that genetically predicted metformin treatment had no effects on PCOS. Sensitivity analyses provided additional confirmation of the reliability of the MR results.

**Conclusions:**

Our two-sample MR analysis did not find genetic evidence supporting a significant association between metformin treatment and PCOS.

## Introduction

Polycystic ovary syndrome (PCOS) is a significant factor underlying female anovulatory infertility, featuring elevated androgen, menstrual irregularities, and polycystic ovaries. Approximately 11–13% of women suffer from PCOS globally [1], which results in a significant health and economic burden worldwide [2]. Despite the high prevalence of PCOS, treatment is often ad hoc and hindered by the limitation of understanding of the etiology and underlying mechanisms of the syndrome.

During the 1980s, a notable correlation between insulin and testosterone levels was discovered in patients with PCOS [3], leading to the eventual acknowledgment of insulin resistance(IR) as a key factor in the pathophysiology of the disease. Consequently, this has prompted extensive research into the potential function of insulin sensitizers, notably metformin, in managing PCOS. Metformin, as a first-line antidiabetic medication for type 2 diabetes(T2D), appears to ameliorate the pathogenesis of PCOS, restore ovarian function, improve the inflammatory state and enhance metabolic status, particularly insulin sensitivity by activating the AMP-activated protein kinase (AMPK) pathway [4]. Positive therapeutic effects are increasingly evident [5,6], encompassing improvements in oligomenorrhea, hirsutism, anovulatory infertility, prevention of pregnancy complications, and obesity. For instance, a meta-analysis of 15 studies with a collective 543 patients diagnosed with PCOS revealed the efficacy of metformin in triggering ovulation [7]. Specifically, the odds ratios (OR) for metformin compared to placebo were 3.88 (CI 2.25 to 6.69), and for the combination of metformin and clomiphene (CC) as opposed to CC alone, the OR was 4.41 (CI 2.37 to 8.22). Another meta-analysis that comprised 12 randomized controlled trials(RCTs) with 608 PCOS women discovered that a 6-month lifestyle intervention plus metformin combination successfully decreased subcutaneous adipose tissue by mean difference(MD) 92.49 cm²(P = 0.01), reduced body mass index(BMI) by MD 0.73 kg/m²(P = 0.0005), and led to an MD improvement of 1.06 in menstrual frequency(P = 0.006), compared with lifestyle ± placebo [8].

However, the American Society for Reproductive Medicine (ASRM) has noted that recommending metformin alone as the primary therapy for ovulation promotion in PCOS is inadequate, and it lacks efficacy for the majority of other indications [9], while the results of current observational studies remain a subject of vivid debate. In particular, live birth rate was considered as the most important outcome, which was demonstrated in a well-designed RCT to be 22.5% in the CC group, 7.2% in the metformin group, and 26.8% in the combination therapy group among 626 infertile women with PCOS [10].Significant differences were observed between metformin compared to CC and combination therapy(P < 0.001), whereas no significant difference was detected between CC and combination therapy (P =  0.31). Yet another multicenter RCT of 320 PCOS women revealed that metformin for 3 months prior to addition of other appropriate infertility treatment raised the likelihood of pregnancy by 1.6 times(CI 1.13 to 2.27) [11]. Furthermore, the effect of metformin in lowering the incidence of miscarriages has also generated debate, which was one of the conclusions of early non-randomized studies involving metformin treatment [12],however, several recent systematic reviews have failed to identify a significant effect of metformin alone or combined with CC on the miscarriage rate [13,14].

Given the above, it is still challenging to elucidate the association between metformin treatment and PCOS due to potential confounders, heterogeneity of PCOS phenotypes and variations in metformin dosage across studies. Mendelian randomization (MR) has recently gained popularity in epidemiological research, and it employs genetic variations as instrumental variables to develop models that explore how exposure elements and diseases are causally related. In contrast to conventional observational studies, MR shows reduces vulnerability to confounding and reverse causality, and it can utilize high-precision genetic sequencing to avoid regression dilution bias caused by measurement errors. This study aimed to evaluate the causal effect of metformin on PCOS in European populations using MR techniques, and provided novel perspectives on PCOS therapy.

## Materials and methods

### Study design

**Fig 1:**
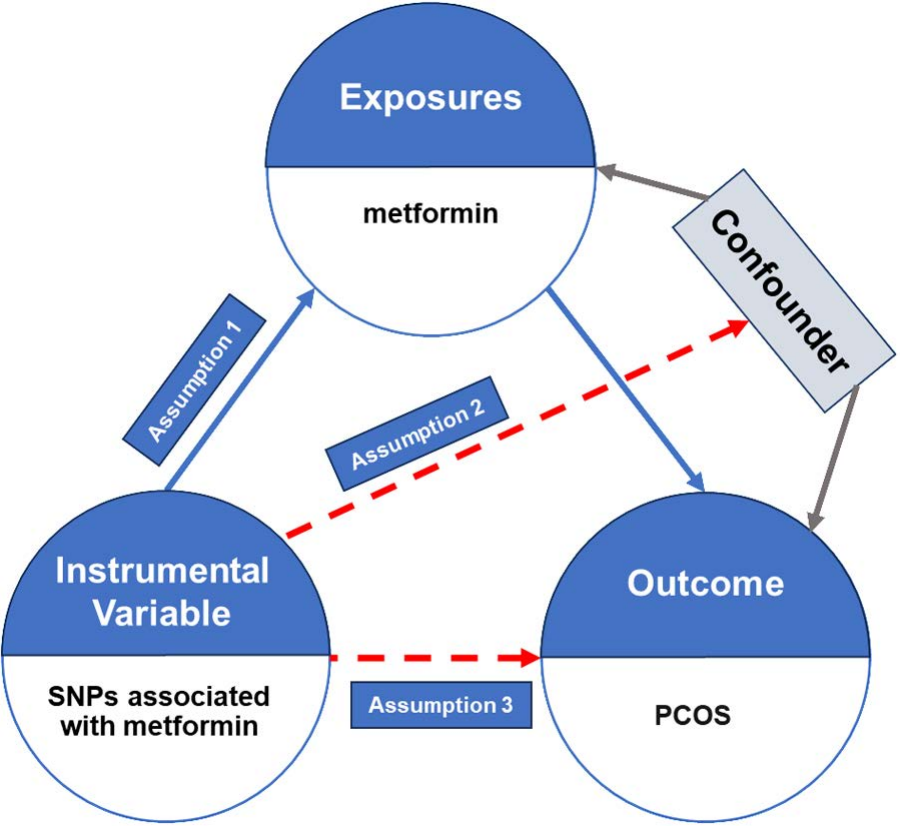
Overall workflow of Mendelian randomization (MR) analysis.

The study’s workflow was depicted in Fig 1. The research conducted a two-sample MR analysis to explored how metformin affected PCOS, adhering to the guidelines outlined in the Strengthening the Reporting of Observational Studies in Epidemiology Using Mendelian Randomization(STROBE-MR) statement. MR analysis is derived from Mendel’s laws of inheritance and founded on three fundamental assumptions. Firstly, the single-nucleotide polymorphisms (SNPs) serving as instrumental variables (IVs) must demonstrate a robust association with the exposure (metformin). Besides, the chosen SNPs must be free from any confounding factors that could potentially bias the results. Lastly, the association between the IVs and the outcome (PCOS) should be mediated solely through the exposure (metformin). These assumptions are critical to ensuring the validity and reliability of MR analysis, allowing for precise estimation of the causal relationship of metformin on PCOS risk.

### Selection of genetic instruments for metformin and PCOS

Genome-wide significant SNPs associated with metformin were obtained from the UK Biobank (UK Biobank, version 3, March 2018), publicly available GWAS data, collecting samples from 500,000 UK individuals (aged 40 to 69). A total of 462,933 individuals, with 11,552 being cases and 451,381 control subjects were sequenced in a European population with Treatment/medication code: metformin (https://gwas.mrcieu.ac.uk/datasets/ukb-b-14609/). Subjects were categorized into case and control groups according to whether or not they took metformin. The included SNPs total 9,851,867. SNPs with p < 5 ×  10^ − 8^ were utilized as instrumental variables in the present study. IVs with an F-statistic of 10 or higher were chosen for further MR analysis.

At the same time, data on PCOS were obtained from a largest GWAS meta-analysis results, which involved 10,074 PCOS cases and 103,164 controls of European descent across seven cohorts (PMID: 30566500), including Rotterdam、UK Biobank、EGCUT、deCODE、Chicago、Boston、23andMe. Diagnosis of PCOS was established either through the National Institutes of Health (NIH) criteria with 2,540 cases and 15,020 controls, the Rotterdam criteria with 2,669 cases and 17,035 controls, or self-reported diagnosis totaling 5,184 cases and 82,759 controls. Data showed that the genetic architecture was the same regardless of the PCOS diagnosis criteria. The NIH criteria mandate hyperandrogenism and ovulatory dysfunction, whereas the Rotterdam criteria involve polycystic ovarian morphology and necessitate the presence of at least two out of the above three traits, leading to four phenotypes. The SNP for PCOS was initially located in the raw data using chromosome and position (chr: pos) and 9295101. We then utilized the in R package “SNPlocs.Hsapiens.dbSNP144.GRCh37” to convert chr: pos to rsID for further analysis. Positions without a rsID and SNP located on mitochondrial or sex chromosomes were removed. After screening, the initial 9,295,102 SNPs were reduced to 8,034,272. We calculated the linkage disequilibrium (LD) to ensure genetic variants in the GWAS databases were independent and that all selected SNPs had r^2^ <  0.01. To mitigate confounding factors, we subsequently examined the potential phenotypes linked to the selected SNPs using the GWAS Catalog and PhenoScanner.

All data for this study were obtained from the publicly available GWAS study database and therefore did not require ethical approval.

### Mendelian randomization analysis

The “TwoSampleMR” R package was used to conduct MR analysis. We employed inverse variance weighted (IVW) as our main approach, complemented by MR Egger, weighted median, simple mode, and weighted mode methods to help make a comprehensive validation of the causal relationship. IVW technique aggregates locus-specific Wald ratio estimates to quantify the effect of genetically anticipated traits on outcomes, assuming every genetic variant is a valid instrument [15]. When there is a portion of IVs may be invalid, the weighted median method offers a more reliable estimate of causality. MR-Egger is carried out to determine if the genetic correlations with the outcome and the risk factor exhibit a dose-response relationship.

### Sensitivity analysis

Within this MR study, we assessed differences among IVs using Cochran’s Q statistic, with a P value exceeding 0.05 indicating the presence of homogeneity. To detect horizontal pleiotropy, which means variants affect outcome via multiple pathways, we employed the MR-Egger intercept test [16]. Additionally, we conducted a leave-one-out sensitivity analysis to ascertain if any individual SNPs could potentially bias the IVW results.

### Statistical analysis

All data analyses were performed using R software (version 4.3.2) and R Studio (version 2023.9.1.494). The R package “ TwoSampleMR (version 0.5.6, https://mrcieu.github.io/TwoSampleMR/)” and its numerous dependencies were utilized for the MR analysis in this study. P-value < 0.05 was required for a difference to be considered statistically significant. In this study, Bonferroni correction was employed to examine the results. Since five methods were used in the MR analysis, the p-value threshold was set at 0.05/5 =  0.01.

## Results

### Genetic variant selection

The GWAS summary statistics utilized in the MR analyses are outlined in Table 1. For the metformin analysis, a total of 32 SNPs were chosen as IVs. The F-statistic of these IVs varied from 31.0899 to 577.9145, with a mean F-statistic of 64.2885 and a median F-statistic of 39.0401. This indicates that all selected SNPs surpassed the conventional threshold of 10, confirming the strength of the IVs and reducing the likelihood of weak instrument bias. The frequency of these IVs ranged from 0.031 to 0.7466.

**Table 1:** The characteristics of SNPs.

SNP	chr	EA	OA	Metformin				PCOS		
				EAF	Beta	SE	p-Value	Beta	SE	p-Value
rs10195252	2	C	T	0.4051	−0.0019	3.00E-04	1.20E-10	−0.0007	0.031	0.98
rs10420309	19	G	A	0.4375	−0.0019	3.00E-04	1.20E-10	0.011	0.033	0.75
rs10965246	9	C	T	0.1767	−0.0043	4.00E-04	2.96E-27	−0.017	0.045	0.71
rs11257655	10	T	C	0.2082	0.0027	4.00E-04	7.39E-12	0.079	0.037	0.034
rs11708067	3	G	A	0.2424	−0.0022	4.00E-04	1.90E-08	0.0079	0.038	0.84
rs13266634	8	T	C	0.3096	−0.0025	4.00E-04	2.05E-10	0.011	0.033	0.74
rs1421085	16	C	T	0.4035	0.0035	3.00E-04	9.43E-32	0.13	0.032	2.00E-05
rs1496653	3	G	A	0.2034	−0.0029	4.00E-04	2.08E-13	0.078	0.038	0.039
rs17036160	3	T	C	0.1175	−0.0031	5.00E-04	2.82E-10	−0.14	0.048	0.0033
rs1800961	20	T	C	0.031	0.0054	9.00E-04	9.87E-10	0.029	0.09	0.75
rs2796441	9	A	G	0.4185	−0.0018	3.00E-04	9.87E-10	−0.0051	0.032	0.87
rs34744311	10	T	C	0.3773	−0.0028	3.00E-04	5.13E-21	0.052	0.032	0.1
rs34872471	10	C	T	0.2918	0.0086	4.00E-04	7.78E-103	−0.015	0.036	0.67
rs459193	5	G	A	0.7466	0.0024	4.00E-04	9.87E-10	0.064	0.036	0.078
rs4686471	3	C	T	0.6101	0.0019	3.00E-04	1.20E-10	−0.0024	0.032	0.94
rs4752792	11	A	G	0.5445	0.0021	3.00E-04	1.28E-12	0.061	0.032	0.055
rs4932264	15	C	T	0.7296	−0.0022	4.00E-04	1.90E-08	−0.072	0.035	0.036
rs67232546	11	T	C	0.2125	0.0023	4.00E-04	4.46E-09	−0.059	0.04	0.13
rs6769511	3	C	T	0.3158	0.0032	3.00E-04	7.29E-27	−0.049	0.033	0.14
rs7177055	15	A	G	0.7175	0.0022	4.00E-04	1.90E-08	0.0088	0.034	0.8
rs72802357	16	T	C	0.0781	−0.004	6.00E-04	1.31E-11	0.093	0.061	0.13
rs73188924	22	A	C	0.2248	0.0022	4.00E-04	1.90E-08	0.029	0.039	0.45
rs7482891	11	G	A	0.6221	−0.0022	3.00E-04	1.12E-13	−0.02	0.033	0.54
rs7756992	6	G	A	0.2664	0.0032	4.00E-04	6.22E-16	0.015	0.035	0.67
rs780093	2	C	T	0.6152	0.0021	3.00E-04	1.28E-12	0	0.032	1
rs849142	7	C	T	0.5051	−0.0024	3.00E-04	6.22E-16	0.018	0.031	0.56
rs8756	12	A	C	0.5176	0.0019	3.00E-04	1.20E-10	0.015	0.031	0.62
rs947791	11	A	G	0.2176	0.0023	4.00E-04	4.46E-09	0.054	0.04	0.17
rs987237	6	G	A	0.1796	0.0024	4.00E-04	9.87E-10	0.011	0.039	0.78
rs9957264	18	A	C	0.1665	−0.0026	4.00E-04	4.02E-11	−0.063	0.041	0.12

SNP: single-nucleotide polymorphism; chr: chromosome; EA: effect allele; OA: other allele; EAF: effect allele frequency.

### Causal effects of metformin treatment on PCOS

**Fig 2:**
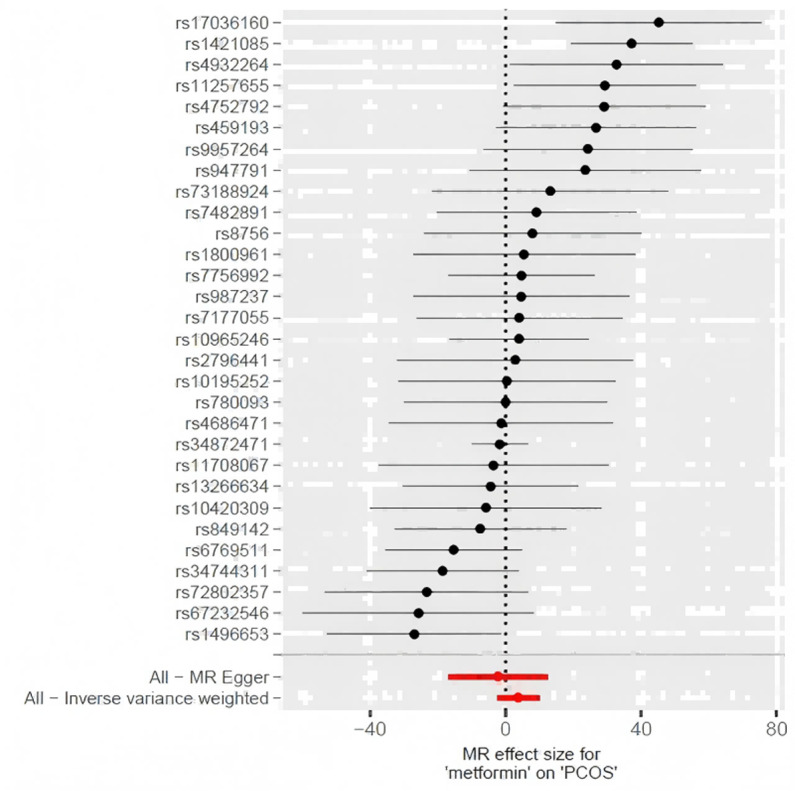
Forest plot of the MR analysis.

**Fig 3:**
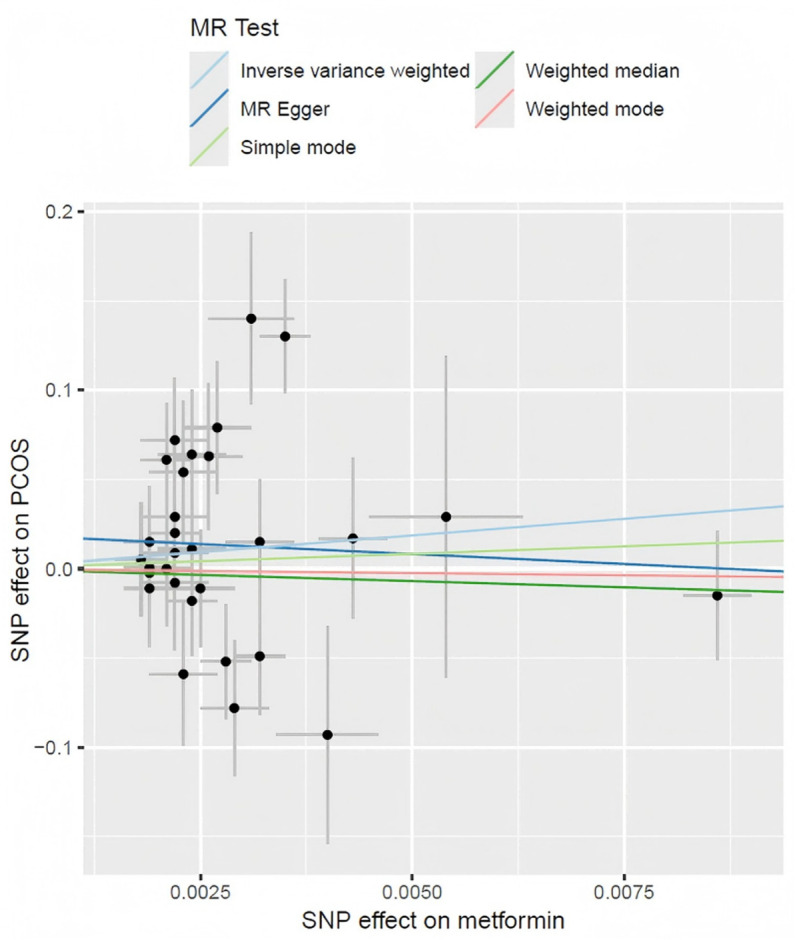
Scatter plot of different MR methods.

Table 2, Fig 2, and Fig 3 present an overview of the MR analysis findings. The study ultimately incorporated a total of 30 IVs. No clear causal relationship was found in the MR studies between PCOS incidence and metformin treatment, as all p-values were greater than 0.05 (p = 0.77 in MR Egger method, p = 0.70 in weighted median method, p = 0.24 in IVW method, p = 0.83 in simple mode method, p = 0.89 in weighted mode method). The confidence intervals of beta and OR in various methods exhibited similar results.

**Table 2:** MR results of the causal association between metformin and PCOS.

Method	Nsnp	Beta	SE	p-Value	LCI	UCI	OR	OR_LCI	OR_UCI
MR Egger	30	−2.22293	7.47531	0.76838	−16.8745	12.42868	0.108292	4.69E-08	249866.1
Weighted median	30	−1.38778	3.679981	0.706088	−8.60054	5.824986	0.24963	0.000184	338.6563
Inverse variance weighted	30	3.723476	3.143393	0.236199	−2.43757	9.884525	41.40807	0.087373	19624.33
Simple mode	30	1.670487	7.769389	0.831265	−13.5575	16.89849	5.314756	1.29E-06	21823306
Weighted mode	30	−0.49238	3.661981	0.89397	−7.66986	6.685105	0.611172	0.000467	800.3948

OR: odds ratio; LCI: lower confidence interval; UCI: upper confidence interval.

### Sensitivity analysis

**Fig 4:**
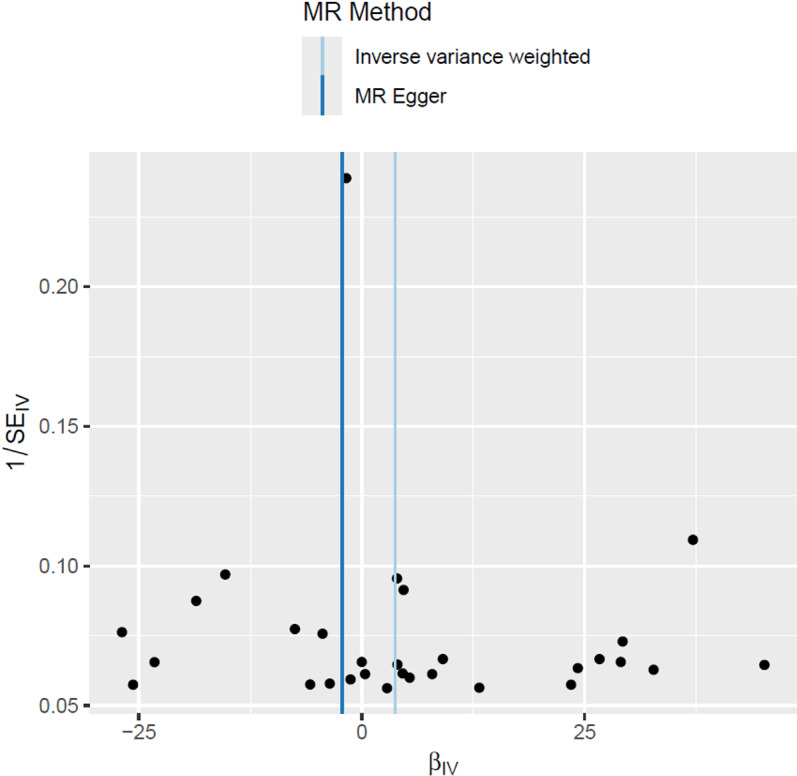
Funnel plot of the MR analysis.

Sensitivity studies were performed in order to assess the consistency and dependability of the research findings. Although a significant degree of SNPs heterogeneity was evaluated by the Cochran’s Q test (Table 3, MR Egger method: Q =  56.51, p =  0.0011; IVW method: Q =  58.07, p =  0.0011), the MR-Egger regression test yielded insufficient evidence of horizontal pleiotropy(MR-Egger intercept =  0.0193; SE =  0.0220; p =  0.3877). Given the relatively large standard error (SE =  0.0220) indicating some degree of uncertainty, the consistency of IVW and MR-Egger reinforced our conclusion that there was no significant evidence of horizontal pleiotropy in the relationship between metformin treatment and PCOS. The symmetry of the funnel plot displayed similar results(Fig 4), suggesting that the analysis was not significantly biased.

**Table 3:** Cochran’s Q test.

Outcome	Exposure	Method	Q	Q_df	Q_pval
PCOS	metformin	MR Egger	56.51323	28	0.001113
PCOS	metformin	Inverse variance weighted	58.06739	29	0.001068

PCOS: polycystic ovary syndrome.

**Fig 5:**
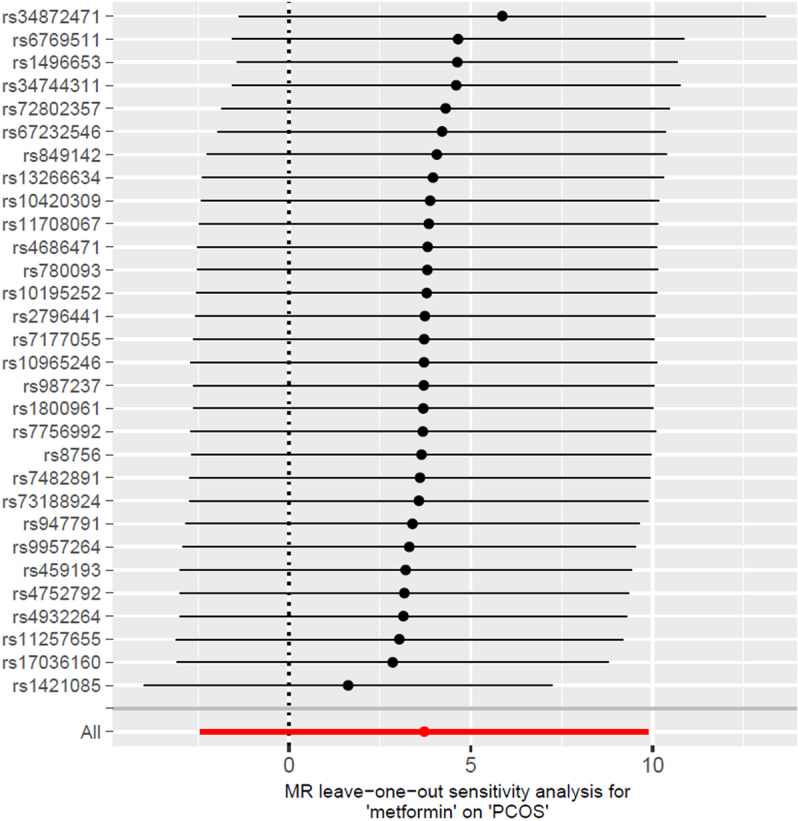
Leave-one-out sensitivity test.

Furthermore, no single SNP was found to significantly alter the causal effect of metformin treatment on PCOS, according to the results of the leave-one-out sensitivity test(Fig 5). Hence, our findings concerning no causal link between metformin treatment and PCOS are robust and dependable.

## Discussion

To the best of our knowledge, our study represents the inaugural MR study investigation into the relationship between PCOS and metformin, with a lack of evidence showing a causal relationship between PCOS risk and genetic predictions of metformin treatment.

Our finding contradicts several previous epidemiological studies, which found protective effects of metformin against PCOS. Two meta-analyses concluded metformin might be beneficial for live birth in women with PCOS subfertility [13,17], while most studies were conducted in China, Turkey, Finland and New Zealand. The average duration of metformin used in the studies was 19.7 weeks, with the median daily dose was 1500mg. The results of the above studies were found varying by BMI. Therefore, it is crucial not to overlook the impact of obesity when analyzing the association between metformin and PCOS.

In this study, all the data used for the MR analysis came from European populations, which may be attributed to the inconsistency with the findings of previous studies. Moreover, though most recent studies use Rotterdam criteria for diagnosing PCOS, other criteria such as NIH criteria and Androgen Excess(AE)-PCOS criteria are still employed. The multiple classification systems have allowed research to be heterogenous with varying characteristics, including oligomenorrhea, hyperandrogenism as well as associated risk factors for cardiovascular disease(obesity, glucose tolerance, etc.), which causes uncertainty regarding the potential benefits of metformin treatment for any given subgroup. Apart from that, recent studies had a significant variation in the doses of metformin, and several studies administered metformin to patients not diagnosed with IR, generally not recommended in clinical practice, which may lead to different effects. In contrast to current studies focusing only on the short-term (usually less than 6 months) effects of metformin intervention, MR analysis can merely reflect the effects of lifetime exposure, which means if there is no continuous influence, the potential effects cannot be explored. Besides, it is also worth noting that causality cannot be confirmed from epidemiological studies, as they are vulnerable to the potential impact of confounding factors or reverse causality [9]. Therefore, larger RCTs with specific phenotypes and dose regimens in a longer follow-up period, and controlled for confounding factors are needed to generate higher quality evidence in the future.

Despite our research showing a lack of causality between metformin and PCOS, it is possible that metformin may influence the progression of the syndrome. A variety of possible pathways linking metformin and PCOS have gained broad acceptance, both at biological and behavioral levels. Adiponectin, an adipocyte-derived hormone exhibiting insulin-sensitizing and anti-inflammatory effects, plays an important role in the pathogenesis of the metabolic syndrome [18]. It has been demonstrated that PCOS patients with obesity, hyperandrogenism, and hyperinsulinism have lower levels of adiponectin [19], though the relationship still remains debatable [20]. Benrick, A. et al. found that adiponectin could protect mice from PCOS-like metabolic phenotypes but had relatively limited protection against reproductive dysfunction in a DHT-induced PCOS-like mouse model [21]. A recent meta-analyse showed metformin treatment was associated with an increase adiponectin levels [22]. Meanwhile, Oróstica ML et al. reported that metformin could restore endometrial cell levels of molecules involved in insulin/adiponectin signaling [23], which might improve the reproductive failures in women with obesity/PCOS. Future research should aim to elucidate whether adiponectin modulation could serve as a therapeutic strategy for PCOS.

Furthermore, low-grade chronic inflammation is increasingly recognized as a key player in the pathophysiology of PCOS, which interlinks obesity, IR, diabetes and cardiovascular disease. A large number of studies have confirmed that patients with PCOS have higher markers of inflammation or their genetic markers, including C-reactive protein (CRP), interleukin 18 (IL-18), tumor necrosis factor (TNF-α), interleukin 6 (IL-6), ferritin and WBC [24]. TNF-α inhibits the tyrosine kinase phosphorylation of the insulin receptor, which results in IR and obesity in PCOS [25]. Long X et al. suggested that elevated endometrial IL-18 mRNA expression in obese PCOS patients might be associated with their risk of implantation failure and miscarriage [26]. Given that hyperinsulinemia may exacerbate health conditions and inflammatory processes in women with PCOS, metformin has been shown to reverse elevated IL-8 levels in PCOS women[i] and is associated with marked reduction in serum CRP [27]. Hu M et al. found that metformin could inhibit the TLR4/IRF-7/NFκB signaling pathways triggered by androgen, thereby suppressing cytokine synthesis and endometrial inflammation in patients with PCOS [28]. Nonetheless, further mechanistic research is required, potentially uncovering novel therapeutic targets.

There are some strengths in our study. First, it is the first time employing MR method to examine the relationship between metformin and PCOS risk, avoiding potential confounding factors and reverse causality. Second, population stratification is not likely to have an effect on our results, as our analysis was limited to individuals with European ancestry. Third, a stringent criterion for selecting instrumental variables was established, utilizing SNPs that were only significantly correlated with metformin treatment and abiding by the three fundamental MR analysis assumptions. Finally, diverse analytical methods were employed, encompassing heterogeneity tests, horizontal pleiotropy assessments, and the leave-one-out analysis, to evaluate the accuracy of the assumption regarding the IVs.

However, our study may have certain limitations. First, our results cannot be used to replace clinical trials in the real world and should be primarily taken as a test of causal relationship. Second, the combined therapeutic effects of drug interactions in clinical settings were not taken into account in our analysis. Third, due to limited availability of data resources, our MR analysis is restricted to the European population, which increases uncertainty whether our results can be applied to other ethnic groups in general. Fourth, heterogeneity was noted in our analysis, whereas our capacity to investigate potential impacts of non-linear connections or variable-related stratification was impeded, such as age, infertility history or baseline hormone levels. Additionally, further stratified analysis might not be allowed to conduct for patients with different clinical phenotypes of PCOS in consideration of limited databases. Therefore, future research should include diverse populations, expand the study samples for various clinical phenotypes of PCOS, and consider both short-term and long-term medication effects.

## Conclusions

To sum up, this MR study did not uncover any causal link between metformin treatment and PCOS. This study presents innovative genetic evidence suggesting that metformin may not be directly involved in the development or management of PCOS. However, further studies are needed to assess the clinical efficacy of metformin in PCOS treatment.

## Supporting information

S1 File Details of single nucleotide polymorphisms related to metformin.
(CSV)

S2 File STROBE checklist.
(DOCX)
