# Supplementary material for: Mendelian randomization study showed no causality between metformin treatment and polycystic ovary syndrome
Source: PLoS One. 2025 Apr 3;20(4):e0321380. doi: 10.1371/journal.pone.0321380 (PMC11967963; doi:10.1371/journal.pone.0321380)
Supplement: S2 File — (DOCX) [file pone.0321380.s002.docx]

**STROBE-MR checklist of recommended items to address in reports of Mendelian randomization studies**^1^ ^2^

| **Item No.** | **Section** | **Checklist item** | **Page No.** | **Relevant text from manuscript** |
| --- | --- | --- | --- | --- |
| 1 | **TITLE and ABSTRACT** | Indicate Mendelian randomization (MR) as the study’s design in the title and/or the abstract if that is a main purpose of the study | 1 | Mendelian randomization study showed no causality between metformin treatment and polycystic ovary syndrome |
|  | **INTRODUCTION** |  |  |  |
| 2 | **Background** | Explain the scientific background and rationale for the reported study. What is the exposure? Is a potential causal relationship between exposure and outcome plausible? Justify why MR is a helpful method to address the study question | 3，4 | For instance, a meta-analysis of 15 studies with a collective 543 patients diagnosed with PCOS revealed the efficacy of metformin in triggering ovulation(6).  However, the American Society for Reproductive Medicine (ASRM) has noted that recommending metformin alone as the primary therapy for ovulation promotion in PCOS is inadequate, and it lacks efficacy for the majority of other indications(8), while the results of current observational studies remain a subject of vivid debate.  Given the above, it is still challenging to elucidate the association between metformin treatment and PCOS due to potential confounders, heterogeneity of PCOS phenotypes and variations in metformin dosage across studies |
| 3 | **Objectives** | State specific objectives clearly, including pre-specified causal hypotheses (if any). State that MR is a method that, under specific assumptions, intends to estimate causal effects | 4，5 | This study utilized a two-sample MR analysis to mimic the prolonged exposure of European populations to metformin, in order to assess its potential association with PCOS and provide novel perspectives on PCOS therapy. |
|  | **METHODS** |  |  |  |
| 4 | **Study design and data sources** | Present key elements of the study design early in the article. Consider including a table listing sources of data for all phases of the study. For each data source contributing to the analysis, describe the following: |  |  |
|  | a) | Setting: Describe the study design and the underlying population, if possible. Describe the setting, locations, and relevant dates, including periods of recruitment, exposure, follow-up, and data collection, when available. | 5 | The research conducted a two-sample MR analysis to explored how metformin affected PCOS, adhering to the guidelines outlined in the Strengthening the Reporting of Observational Studies in Epidemiology Using Mendelian Randomization(STROBE-MR) statement. MR analysis is derived from Mendel's laws of inheritance and founded on three fundamental assumptions. |
|  | b) | Participants: Give the eligibility criteria, and the sources and methods of selection of participants. Report the sample size, and whether any power or sample size calculations were carried out prior to the main analysis | 6 | Genome-wide significant SNPs associated with metformin were identified from a large-scale GWAS involving 462,933 individuals, with 11,552 being cases and 451,381 control subjects of European descent sourced from the UK Biobank (<https://gwas.mrcieu.ac.uk/datasets/ukb-b-14609/>).  At the same time, data on PCOS were obtained from a GWAS involving 10,074 PCOS cases and 103,164 controls of European descent across seven cohorts (PMID: 30566500). |
|  | c) | Describe measurement, quality control and selection of genetic variants | 6 | Genome-wide significant SNPs associated with metformin were identified from a large-scale GWAS involving 462,933 individuals, with 11,552 being cases and 451,381 control subjects of European descent sourced from the UK Biobank (https://gwas.mrcieu.ac.uk/datasets/ukb-b-14609/). Subjects were categorized into case and control groups according to whether or not they took metformin. The included SNPs total 9,851,867. SNPs with p< 5 × 10−8 were utilized as instrumental variables in the present study. IVs with an F-statistic of 10 or higher were chosen for further MR analysis. |
|  | d) | For each exposure, outcome, and other relevant variables, describe methods of assessment and diagnostic criteria for diseases | 6 | At the same time, data on PCOS were obtained from a GWAS involving 10,074 PCOS cases and 103,164 controls of European descent across seven cohorts (PMID: 30566500). Diagnosis of PCOS was established either through the National Institutes of Health (NIH) criteria with 2,540 cases and 15,020 controls, the Rotterdam criteria with 2,669 cases and 17,035 controls, or self-reported diagnosis totaling 5,184 cases and 82,759 controls. The NIH criteria mandate hyperandrogenism and ovulatory dysfunction, whereas the Rotterdam criteria involve polycystic ovarian morphology and necessitate the presence of at least two out of the above three traits, leading to four phenotypes. |
|  | e) | Provide details of ethics committee approval and participant informed consent, if relevant |  | Not relevant |
| 5 | **Assumptions** | Explicitly state the three core IV assumptions for the main analysis (relevance, independence and exclusion restriction) as well assumptions for any additional or sensitivity analysis | 5 | Firstly, the single-nucleotide polymorphisms (SNPs) serving as instrumental variables (IVs) must demonstrate a robust association with the exposure (metformin). Besides, the chosen SNPs must be free from any confounding factors that could potentially bias the results. Lastly, the association between the IVs and the outcome (PCOS) should be mediated solely through the exposure (metformin). These assumptions are critical to ensuring the validity and reliability of MR analysis, allowing for precise estimation of the causal relationship of metformin on PCOS risk. |
| 6 | **Statistical methods: main analysis** | Describe statistical methods and statistics used |  |  |
|  | a) | Describe how quantitative variables were handled in the analyses (i.e., scale, units, model) |  | No quantitative variables in this study. |
|  | b) | Describe how genetic variants were handled in the analyses and, if applicable, how their weights were selected |  | SNPs with p< 5 × 10−8 were utilized as instrumental variables in the present study. IVs with an F-statistic of 10 or higher were chosen for further MR analysis.  After screening, the initial 9,295,102 SNPs were reduced to 8,034,272. Linkage disequilibrium (LD) was assessed to prevent bias resulting from LD. To mitigate confounding factors, we subsequently examined the potential phenotypes linked to the selected SNPs using the GWAS Catalog and PhenoScanner. |
|  | c) | Describe the MR estimator (e.g. two-stage least squares, Wald ratio) and related statistics. Detail the included covariates and, in case of two-sample MR, whether the same covariate set was used for adjustment in the two samples | 7 | The “TwoSampleMR” R package was used to conduct MR analysis. We employed inverse variance weighted (IVW) as our main approach, complemented by MR Egger, weighted median, simple mode, and weighted mode methods to help make a comprehensive validation of the causal relationship. IVW technique aggregates locus-specific Wald ratio estimates to quantify the effect of genetically anticipated traits on outcomes, assuming every genetic variant is a valid instrument. When there is a portion of IVs may be invalid, the weighted median method offers a more reliable estimate of causality. MR-Egger is carried out to determine if the genetic correlations with the outcome and the risk factor exhibit a dose-response relationship. |
|  | d) | Explain how missing data were addressed |  | No missing data. |
|  | e) | If applicable, indicate how multiple testing was addressed |  | Not applicable. |
| 7 | **Assessment of assumptions** | Describe any methods or prior knowledge used to assess the assumptions or justify their validity | 7 | Within this MR study, we assessed differences among IVs using Cochran’s Q statistic, with a P value exceeding 0.05 indicating the presence of homogeneity. To detect horizontal pleiotropy, we employed the MR-Egger intercept test. Additionally, we conducted a leave-one-out sensitivity analysis to ascertain if any individual SNPs could potentially bias the IVW results. |
| 8 | **Sensitivity analyses and additional analyses** | Describe any sensitivity analyses or additional analyses performed (e.g. comparison of effect estimates from different approaches, independent replication, bias analytic techniques, validation of instruments, simulations) | 7 | Within this MR study, we assessed differences among IVs using Cochran’s Q statistic, with a P value exceeding 0.05 indicating the presence of homogeneity. To detect horizontal pleiotropy, we employed the MR-Egger intercept test. Additionally, we conducted a leave-one-out sensitivity analysis to ascertain if any individual SNPs could potentially bias the IVW results. |
| 9 | **Software and pre-registration** |  |  |  |
|  | a) | Name statistical software and package(s), including version and settings used | 7 | All data analyses were performed using R software (version 4.3.2) and R Studio (version 2023.9.1.494). The R package " TwoSampleMR (version 0.5.6, https://mrcieu.github.io/TwoSampleMR/)" and its numerous dependencies were utilized for the MR analysis in this study. |
|  | b) | State whether the study protocol and details were pre-registered (as well as when and where) |  | The study needed no registration. |
|  | **RESULTS** |  |  |  |
| 10 | **Descriptive data** |  |  |  |
|  | a) | Report the numbers of individuals at each stage of included studies and reasons for exclusion. Consider use of a flow diagram | 6,8 | We then utilized the in R package “SNPlocs.Hsapiens.dbSNP144.GRCh37” to convert chr: pos to rsID for further analysis. Positions without a rsID and SNP located on mitochondrial or sex chromosomes were removed. After screening, the initial 9,295,102 SNPs were reduced to 8,034,272. Linkage disequilibrium (LD) was assessed to prevent bias resulting from LD. To mitigate confounding factors, we subsequently examined the potential phenotypes linked to the selected SNPs using the GWAS Catalog and PhenoScanner.  For the metformin analysis, a total of 32 SNPs were chosen as IVs. |
|  | b) | Report summary statistics for phenotypic exposure(s), outcome(s), and other relevant variables (e.g. means, SDs, proportions) | 8 | For the metformin analysis, a total of 32 SNPs were chosen as IVs. The F-statistic of these IVs varied from 31.0899 to 577.9145. The frequency of these IVs ranged from 0.031 to 0.7466. |
|  | c) | If the data sources include meta-analyses of previous studies, provide the assessments of heterogeneity across these studies |  | Not meta-analyses included |
|  | d) | For two-sample MR:  i.  Provide justification of the similarity of the genetic variant-exposure associations between the exposure and outcome samples  ii.  Provide information on the number of individuals who overlap between the exposure and outcome studies | 6,8 | The included SNPs total 9,851,867. SNPs with p< 5 × 10−8 were utilized as instrumental variables in the present study. IVs with an F-statistic of 10 or higher were chosen for further MR analysis.  For the metformin analysis, a total of 32 SNPs were chosen as IVs. The F-statistic of these IVs varied from 31.0899 to 577.9145. The frequency of these IVs ranged from 0.031 to 0.7466. |
| 11 | **Main results** |  |  |  |
|  | a) | Report the associations between genetic variant and exposure, and between genetic variant and outcome, preferably on an interpretable scale | 8 | No clear causal relationship was found in the MR studies between PCOS incidence and metformin treatment, as all p-values were greater than 0.05 (p=0.77 in MR Egger method, p=0.70 in weighted median method, p=0.24 in IVW method, p=0.83 in simple mode method, p=0.89 in weighted mode method). The confidence intervals of beta and OR in various methods exhibited similar results. |
|  | b) | Report MR estimates of the relationship between exposure and outcome, and the measures of uncertainty from the MR analysis, on an interpretable scale, such as odds ratio or relative risk per SD difference | 19 | Table2 |
|  | c) | If relevant, consider translating estimates of relative risk into absolute risk for a meaningful time period |  | Not relevant |
|  | d) | Consider plots to visualize results (e.g. forest plot, scatterplot of associations between genetic variants and outcome versus between genetic variants and exposure) | 17 | Figure2-4 |
| 12 | **Assessment of assumptions** |  |  |  |
|  | a) | Report the assessment of the validity of the assumptions | 8 | Table 2, Figure 2, and Figure 3 present an overview of the MR analysis findings.  The confidence intervals of beta and OR in various methods exhibited similar results. |
|  | b) | Report any additional statistics (e.g., assessments of heterogeneity across genetic variants, such as *I^2^*, Q statistic or E-value) | 19 | Table3 |
| 13 | **Sensitivity analyses and additional analyses** |  |  |  |
|  | a) | Report any sensitivity analyses to assess the robustness of the main results to violations of the assumptions | 8 | Sensitivity studies were performed in order to assess the consistency and dependability of the research findings. A significant degree of SNPs heterogeneity was evaluated by the Cochran’s Q test (Table 3, MR Egger method: Q = 56.51, p = 0.0011; IVW method: Q = 58.07, p = 0.0011). Nevertheless the MR-Egger regression test yielded insufficient evidence of horizontal pleiotropy(MR-Egger intercept = 0.0193; SE = 0.0220; p = 0.3877). Similar results were displayed by the funnel plot(Figure 4). |
|  | b) | Report results from other sensitivity analyses or additional analyses | 9 | Furthermore, no single SNP was found to significantly alter the causal effect of metformin treatment on PCOS, according to the results of the leave-one-out sensitivity test(Figure 5). Hence, our findings concerning no causal link between metformin treatment and PCOS are robust and dependable. |
|  | c) | Report any assessment of direction of causal relationship (e.g., bidirectional MR) | 8 | A significant degree of SNPs heterogeneity was evaluated by the Cochran’s Q test (Table 3, MR Egger method: Q = 56.51, p = 0.0011; IVW method: Q = 58.07, p = 0.0011). Nevertheless the MR-Egger regression test yielded insufficient evidence of horizontal pleiotropy(MR-Egger intercept = 0.0193; SE = 0.0220; p = 0.3877). |
|  | d) | When relevant, report and compare with estimates from non-MR analyses |  | Not relevant |
|  | e) | Consider additional plots to visualize results (e.g., leave-one-out analyses) | 17 | Figure5 |
|  | **DISCUSSION** |  |  |  |
| 14 | **Key results** | Summarize key results with reference to study objectives | 9 | To the best of our knowledge, our study represents the inaugural MR study investigation into the relationship between PCOS and metformin, with a lack of evidence showing a causal relationship between PCOS risk and genetic predictions of metformin treatment. |
| 15 | **Limitations** | Discuss limitations of the study, taking into account the validity of the IV assumptions, other sources of potential bias, and imprecision. Discuss both direction and magnitude of any potential bias and any efforts to address them | 12 | However, our study may have certain limitations. First, our results cannot be used to replace clinical trials in the real world and should be primarily taken as a test of causal relationship. Second, the combined therapeutic effects of drug interactions in clinical settings were not taken into account in our analysis. Third, due to limited availability of data resources, our MR analysis is restricted to the European population, which increases uncertainty whether our results can be applied to other ethnic groups in general. Fourth, heterogeneity was noted in our analysis, whereas our capacity to investigate potential impacts of non-linear connections or variable-related stratification was impeded, such as age, infertility history or baseline hormone levels. Additionally, further stratified analysis might not be allowed to conduct for patients with different clinical phenotypes of PCOS in consideration of limited databases. Therefore, future research should include diverse populations, expand the study samples for various clinical phenotypes of PCOS, and consider both short-term and long-term medication effects. |
| 16 | **Interpretation** |  |  |  |
|  | a) | Meaning: Give a cautious overall interpretation of results in the context of their limitations and in comparison with other studies | 10 | In this study, all the data used for the MR analysis came from European populations, which may be attributed to the inconsistency with the findings of previous studies. Moreover, though most recent studies use Rotterdam criteria for diagnosing PCOS, other criteria such as NIH criteria and Androgen Excess(AE)-PCOS criteria are still employed. The multiple classification systems have allowed research to be heterogenous with varying characteristics.  Apart from that, recent studies had a significant variation in the doses of metformin, and several studies administered metformin to patients not diagnosed with IR, generally not recommended in clinical practice, which may lead to different effects. In contrast to current studies focusing only on the short-term (usually less than 6 months) effects of metformin intervention, MR analysis can merely reflect the effects of lifetime exposure, which means if there is no continuous influence, the potential effects cannot be explored. |
|  | b) | Mechanism: Discuss underlying biological mechanisms that could drive a potential causal relationship between the investigated exposure and the outcome, and whether the gene-environment equivalence assumption is reasonable. Use causal language carefully, clarifying that IV estimates may provide causal effects only under certain assumptions | 11 | Despite our research showing a lack of causality between metformin and PCOS, it is possible that metformin may influence the progression of the syndrome. A variety of possible pathways linking metformin and PCOS have gained broad acceptance, both at biological and behavioral levels. It has been shown that metformin not only improved weight and metabolic disorders, but also enhanced ovarian dysfunction, possibly by regulating ferroptosis through the SIRT3/AMPK/mTOR pathway in a PCOS model of mice developed by letrozole in conjunction with a high-fat diet(17). Furthermore, metformin could inhibit the TLR4/IRF-7/NFκB signaling pathways triggered by androgen, thereby suppressing cytokine synthesis and endometrial inflammation in patients with PCOS(18). In addition, metformin could exert beneficial effect on leukocyte/endothelium interactions by reducing polymorphonuclear(PMN) rolling flux, as well as levels of intercellular cell adhesion molecule 1(ICAM-1), selectins, interleukin 6(IL-6), and tumor necrosis factor alpha(TNF-α) in serum, potentially lowering the chances of vascular events in patients with PCOS(19). |
|  | c) | Clinical relevance: Discuss whether the results have clinical or public policy relevance, and to what extent they inform effect sizes of possible interventions | 10 | Therefore, larger RCTs with specific phenotypes and dose regimens in a longer follow-up period, and controlled for confounding factors are needed to generate higher quality evidence in the future. |
| 17 | **Generalizability** | Discuss the generalizability of the study results (a) to other populations, (b) across other exposure periods/timings, and (c) across other levels of exposure | 10，12 | In contrast to current studies focusing only on the short-term (usually less than 6 months) effects of metformin intervention, MR analysis can merely reflect the effects of lifetime exposure, which means if there is no continuous influence, the potential effects cannot be explored.  Second, the combined therapeutic effects of drug interactions in clinical settings were not taken into account in our analysis. Third, due to limited availability of data resources, our MR analysis is restricted to the European population, which increases uncertainty whether our results can be applied to other ethnic groups in general. |
|  | **OTHER INFORMATION** |  |  |  |
| 18 | **Funding** | Describe sources of funding and the role of funders in the present study and, if applicable, sources of funding for the databases and original study or studies on which the present study is based | 13 | This work was supported by the National Natural Science Foundation of China (No. 21737001). |
| 19 | **Data and data sharing** | Provide the data used to perform all analyses or report where and how the data can be accessed, and reference these sources in the article. Provide the statistical code needed to reproduce the results in the article, or report whether the code is publicly accessible and if so, where | 13 | The original contributions presented in this study are included in the article, further inquires can be directed to the corresponding author.1 |
| 20 | **Conflicts of Interest** | All authors should declare all potential conflicts of interest | 13 | The authors declare that the research was conducted in the absence of any commercial or financial relationships that could be construed as a potential conflict of interest. |

This checklist is copyrighted by the Equator Network under the Creative Commons Attribution 3.0 Unported (CC BY 3.0) license.

1. Skrivankova VW, Richmond RC, Woolf BAR, Yarmolinsky J, Davies NM, Swanson SA, et al. Strengthening the Reporting of Observational Studies in Epidemiology using Mendelian Randomization (STROBE-MR) Statement. JAMA. 2021;under review.

2. Skrivankova VW, Richmond RC, Woolf BAR, Davies NM, Swanson SA, VanderWeele TJ, et al. Strengthening the Reporting of Observational Studies in Epidemiology using Mendelian Randomisation (STROBE-MR): Explanation and Elaboration. BMJ. 2021;375:n2233.
